# The New Webster

**Published:** 1869-02

**Authors:** J. H. Raymond


The New Webster is glorious—it is perfect—it distances and defies competition—it leaves nothing to be desired. As a monument of literary labor, or as a business enterprise, magnificent in conception and almost faultless in execution, I think it equally admirable; and if you should die to-morrow, you may feel that, so far as earthly honor is concerned, your monument is built. But I can not doubt that a grateful country will appreciate the immense service you have rendered to the national language, scholarship and reputation by this great work, and in due time render you an adequate reward.—*J. H. Raymond, LL. D.*

